# Molecular magnetic resonance imaging in cancer

**DOI:** 10.1186/s12967-015-0659-x

**Published:** 2015-09-23

**Authors:** Mohammad Haris, Santosh K. Yadav, Arshi Rizwan, Anup Singh, Ena Wang, Hari Hariharan, Ravinder Reddy, Francesco M. Marincola

**Affiliations:** Sidra Medical and Research Center, 26999, Doha, Qatar; All India Institute of Medical Sciences, New Delhi, India; Center for Biomedical Engineering, Indian Institute of Technology, Delhi, India; Center for Magnetic Resonance and Optical Imaging, Department of Radiology, University of Pennsylvania, Philadlephia, PA 19104 USA

**Keywords:** Cancer, Magnetic resonance imaging, Magnetic resonance spectroscopy, Hyperpolarization, Chemical exchange saturation transfer (CEST) imaging, Reporter genes, Cancer immunotherapy

## Abstract

The ability to identify key biomolecules and molecular changes associated with cancer malignancy and the capacity to monitor the therapeutic outcome against these targets is critically important for cancer treatment. Recent developments in molecular imaging based on magnetic resonance (MR) techniques have provided researchers and clinicians with new tools to improve most facets of cancer care. Molecular imaging is broadly described as imaging techniques used to detect molecular signature at the cellular and gene expression levels. This article reviews both established and emerging molecular MR techniques in oncology and discusses the potential of these techniques in improving the clinical cancer care. It also discusses how molecular MR, in conjunction with other structural and functional MR imaging techniques, paves the way for developing tailored treatment strategies to enhance cancer care.

## Background

Advancement in understanding of molecular and cellular processes in cancer led to the development of various imaging based techniques, which can monitor these processes non-invasively in vivo and provide opportunity to better describe cancer biology, and to assess therapeutic targets. Imaging techniques which target these molecular and cellular physiologies are grouped under a broad term “molecular imaging”. Molecular imaging provides a new approach to image and characterize the key biomolecules and molecular changes associated with the cancer malignancy [1]. Molecular Imaging offers non-invasive and repetitive detection of cancer cellular-biochemistry and physiology in vivo, which may help to predict the tumor response against a specific treatment and may provide more definite criteria for patient selection to identify those that would respond to treatment.

Various imaging modalities including single photon emission computed tomography (SPECT), positron emission tomography (PET), optical imaging, magnetic resonance imaging (MRI), and magnetic resonance spectroscopy (MRS) have been widely used to monitor structural, functional, and molecular changes in cancer tissues both clinically and pre-clinically [2–12]. PET and SPECT use radiotracers to image and measure the biological activity at targeted site, and are generally considered as molecular imaging modalities. However, despite exquisite sensitivity they are beset by poor resolution and the application of nuclear radiation may preclude their use for repetitive measurements in a short time period. Optical imaging has been used to image specific molecular features of cancer by employing molecular targeted contrast agents [7, 13]. Studies have suggested that optical based method can provide early information of treatment efficacy [14, 15]. However, requirement of the optical probe insertion in tissue limits its repetitive use, and also it is not suitable for studying parts of tissue that are distant from the probe.

Due to its non-invasive characteristics and high spatial resolution, MRI is one of the most powerful imaging tools available in diagnostic imaging, and has been readily used in preclinical research studies too. Recent development of new MR methods, which focus on imaging of molecular signatures, and development of novel molecular contrast agents have expanded the strength of MRI in characterizing tissue physiological and molecular changes. In this review article, we outline different molecular MR imaging techniques describing cellular and molecular changes in cancer, and their roles in cancer prognosis, staging and monitoring therapeutic efficacy.

## Review

### Imaging of cancer metabolism

Altered cellular metabolism is key for cancer growth and malignancy [16, 17]. Many of the biochemical pathways particularly, glycolysis, pentose phosphate pathway (PPP), and TCA cycle are subjected to alternative regulation in cancer cells [18–23]. Monitoring and understanding the cancer metabolism in vivo drastically improves diagnosis and treatment planning of cancer. It was Otto Warburg who demonstrated high glucose consumption and lactate production in cancer compared to healthy tissues and conceptualized that tumor metabolism differs from that of normal tissue [24, 25]. This pivotal observation created a field of tumor metabolism, and led to the development of different MR techniques to monitor metabolic changes in cancer tissues in vivo.

### Magnetic resonance spectroscopy (MRS)

Magnetic resonance spectroscopy has been widely used to detect metabolic changes in cancerous as well as in normal tissues [26]. Different metabolic markers, detectable by MRS, not only provide information on biochemical changes in response to tumor growth but also delineate different metabolic tumor phenotypes. Proton MRS (^1^H MRS) is widely used MRS method to monitor metabolic changes in cancer tissue [27–32]. The other active nuclei such as, ^31^P (phosphorus), ^13^C (carbon) and ^19^F (fluorine) are also being used to monitor bioenergetics and metabolic flux in cancer [33–46].

^1^H MRS is most commonly used method to detect metabolic changes in cancer tissues [27–32]. The ^1^H signal from total choline (Cho) is significantly elevated in cancer tissue, which is shown to be correlated with cellular proliferation in cancer [47–50]. Choline ratios with other metabolites are routinely used to classify cancer aggressiveness. For example- Cho/(N-acetylaspartate (NAA)) ratio is used to distinguish low and high grade astrocytoma’s and gliomas [51], while Cho**/**(creatine) Cr is used to differentiate low grade glioma from benign lesion [52]. It has been shown that distinct patterns of Cho metabolism are associated with different gene expression profiles in the luminal and basal like breast cancers xenograft models [53], and the serial choline levels measured by MRS provide an early indicator of treatment response in the breast cancer [54]. Alteration in the profile of choline compounds is associated with the malignant transformation of breast, and ovarian cancers [55–57]. Both breast and ovarian cancer cells showed higher phosphocholine (PC) signal while glycero-PC (GPC) signal was predominant in nonmalignant breast and ovarian epithelial cells [56, 58]. Not only ^1^H MRS but ^31^P MRS also has been used to detect the changes in choline metabolites such as phosphomonoesters, and phosphodiesters in cancer, which has been proven valuable in monitoring response of tumors to anti-cancer therapy.

Magnetic resonance spectroscopic imaging (MRSI) provides spatial mapping of endogenous metabolites and thus can provide heterogeneous distribution of these metabolites in cancer tissue [59, 60]. The spatial mapping of Cho proton signal can reveal aggressive areas in tumor tissue and help to monitor therapeutic responses [61, 62]. In vivo MRSI improves diagnostic specificity of malignant human cancers and is becoming an important clinical tool for cancer’s management and care [63].

^31^P MRS, in spite of its lower sensitivity provides important information about cancer metabolism. ^31^P MRS detects metabolites such as phosphocreatine (PCr), nucleotide triphosphate (NTP), phosphomonoesters (PME), phosphodiesters (PDE) and inorganic phosphate (Pi) [64]. Phosphomonoesters include phosphocholine, phosphoethanolamine and phosphoserine, while phosphodiesters include glycerylphosphocholine, glycerophosphoethanolamine and glycerylphosphoserine. In breast cancer, ^31^P MRS showed increased concentration of PME compared to normal breast tissue [65] and following chemotherapy a decrease in the PME level was observed [66, 67]. Study on breast cancer cell lines showed higher phosphocholine in malignant cell line compared to nonmalignant cell line [58]. It has been shown that in some cancers malignant progression is associated with a switch from GPC to PC [57]. Elevated level of both PC and GPC has been detected in prostate cancer cells [68]. PME/PDE ratios predicted early response to chemotherapy in patients with soft tissue sarcomas [69]. In addition, ^31^P MRS studies have also showed that the ratio of PCr/Pi and NTP/Pi is correlated with the tumor oxygen level [70, 71].

### Hyperpolarized MRI in cancer

Techniques using hyperpolarized ^13^C labeled pyruvate infusion to monitor increased glycolysis in cancer have the potential to improve the way MRI is used for detection and characterization of cancer. To date, ^13^C pyruvate has been the most widely used hyperpolarize substrate both in preclinical and clinical studies [72, 73]. Level of hyperpolarized [^1–13^C] lactate following intravenous injection of ^13^C pyruvate increases with cancer progression and reduces after therapy [74]. Flux of hypepolarized^13^C between pyruvate and lactate has been used to visualize prostate cancer [75]. Hyperpolarized ^13^C lactate study in a transgenic prostate cancer mouse model showed that the hyperpolarized lactate level progressively increased with cancer aggressiveness and correlated significantly with histological grading [72]. Studies with lymphoma-bearing mice injected with hyperpolarized [^1–13^C] pyruvate have shown lower rate of pyruvate to lactate conversion after onset of chemotherapy, which correlated with the amount of cell death caused by the chemotherapeutic drug [76].

**Fig. 1:**
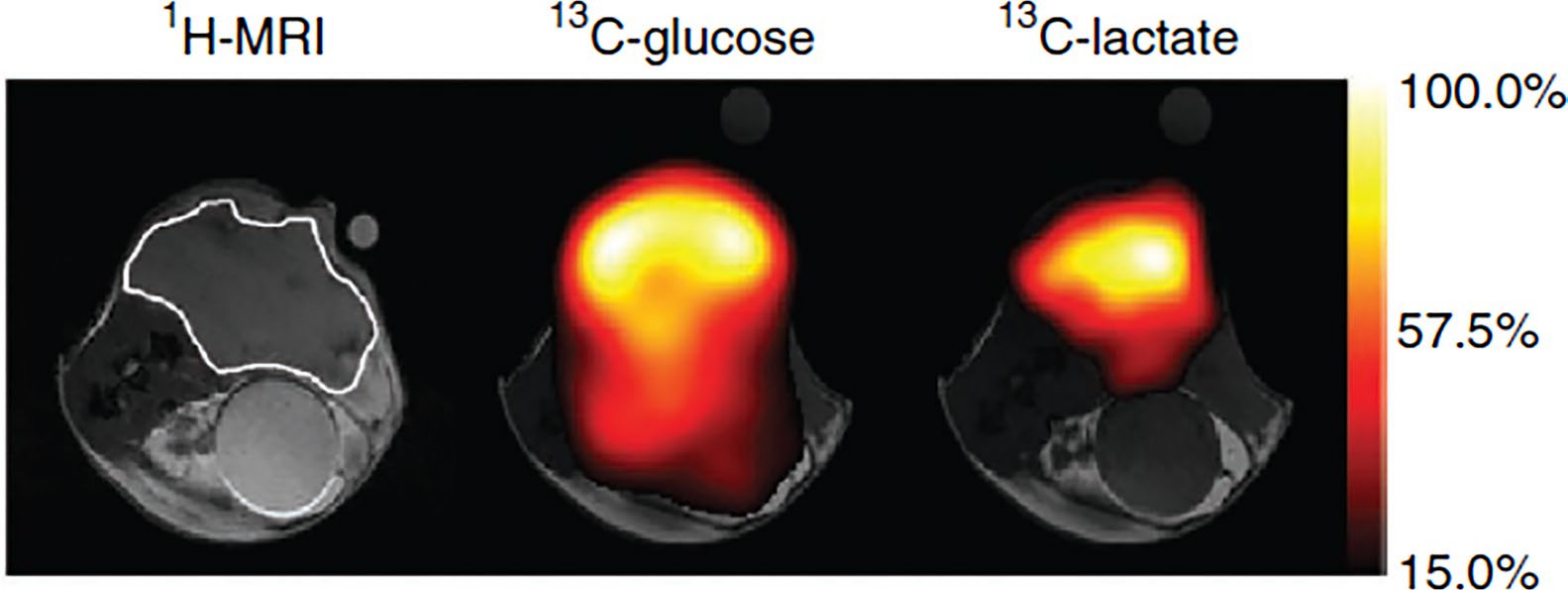
Anatomical image is showing a subcutaneous EL4 tumor in a mouse model. Chemical-shift imaging for ^13^C-glucose and ^13^C-lactate from the same animal was obtained 15 s after intravenous injection of 0.4 mL of 200 mM hyperpolarized glucose. ^13^C-lactate signal demonstrates generation of lactate through anaerobic glycosylation. This material was reproduced with permission from the Nature Publishing Group and Rodrigues et al. [77]

Recently, hyperpolarized ^13^C glucose has been used to monitor the glycolytic flux in mouse lymphoma tumor model [77] (Fig. 1). Despite the short spin–lattice **(**T_1_**)** relaxation of carbon in glucose ^13^C lactate signal was measured within tumor, which decreases following chemotherapeutic drug treatment. In addition, signal from 6-phosphogluconate was detected which is generated through the PPP. However, sensitivity of detection of hyperpolarized ^13^C lactate with hyperpolarized ^13^C glucose infusion was much lower than hyperpolarized ^13^C lactate detected following injection of hyperpolarized ^13^C pyruvate. Nonetheless, this method could provide a new way to monitor the glycolytic flux and PPP activity in tumor.

**Fig. 2:**
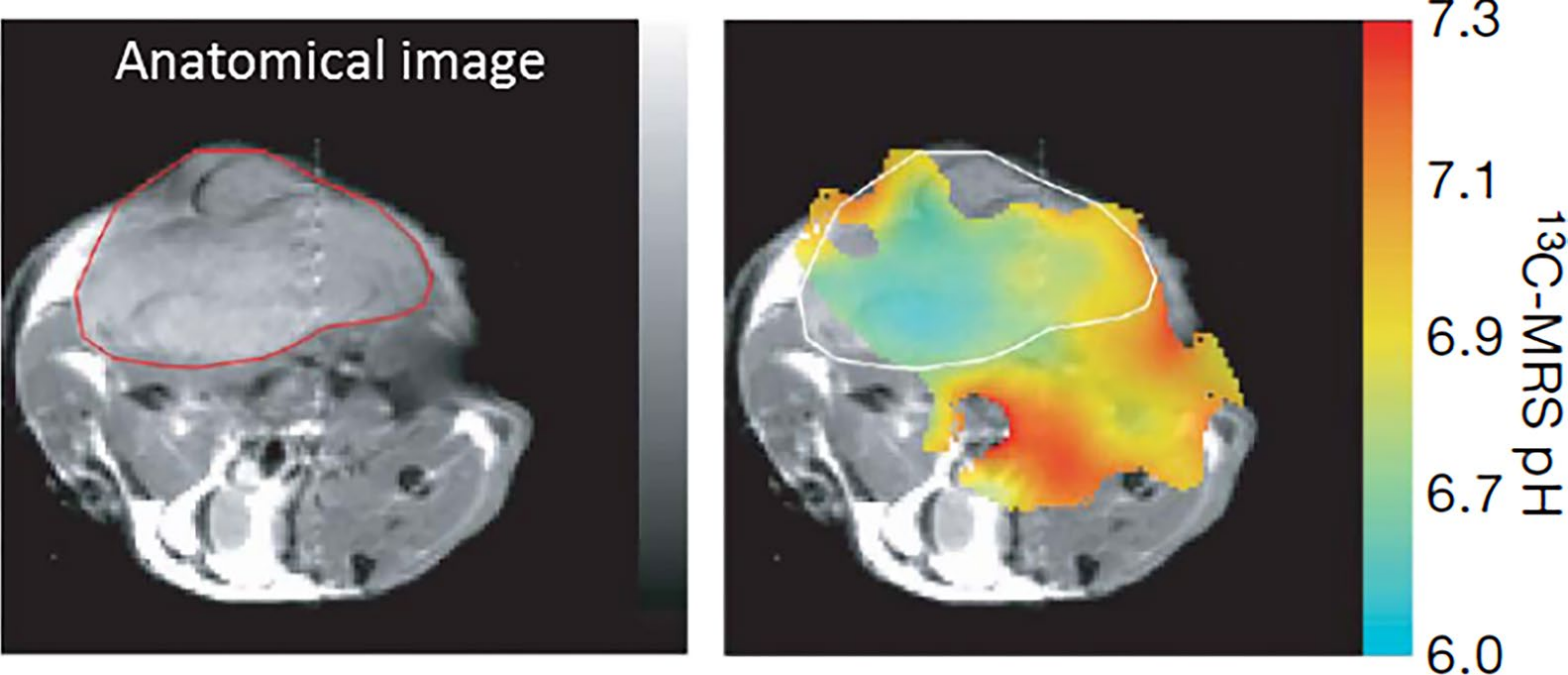
pH mapping of tumor tissue. Anatomical image is showing a subcutaneously implanted EL4 tumor. The pH map generated by calculating the ratio of the hyperpolarized H^13^CO3 and ^13^CO2 voxel intensities using handersal-hasselbatch equation. Reproduced with permission from the Nature Publishing Group and Gallagher et al. [78]

In a recent study, it has been shown that the tissue pH can be mapped through hyperpolarized MRI [78]. Since many pathological changes are associated with pH changes, monitoring the pH in vivo can provide useful information about the tissue pathological stage. The pH map can be generated noninvasively by targeting conversion of the injected hyperpolarized ^13^C labeled bicarbonate to hyperpolarized carbon dioxide in tumor tissues [78] (Fig. 2). The mapping of pH changes in tumor can be used as a marker for evaluating the treatment efficacy as well as in designing of the effective cancer treatment protocols.

In clinic, by using hyperpolarized technique the treatment response can be monitored within hours, and based on the observation more effective treatment could be initiated.

### Chemical exchange saturation transfer (CEST) imaging

Chemical exchange saturation transfer (CEST) is a new contrast enhancement technique that enables the indirect detection of molecules and macromolecules possessing exchangeable protons [79–81]. In CEST experiment, these exchangeable protons can be specifically saturated using frequency selective radiofrequency (RF) saturation pulse which leads to the zero magnetization on these exchangeable protons, and their exchange with the bulk water protons decrease the bulk water signal [80, 81]. The decrease in bulk water signal can be quantified and mapped to provide high resolution images of specific molecules and macromolecules.

### Imaging of mobile protein and peptide in cancer

**Fig. 3:**
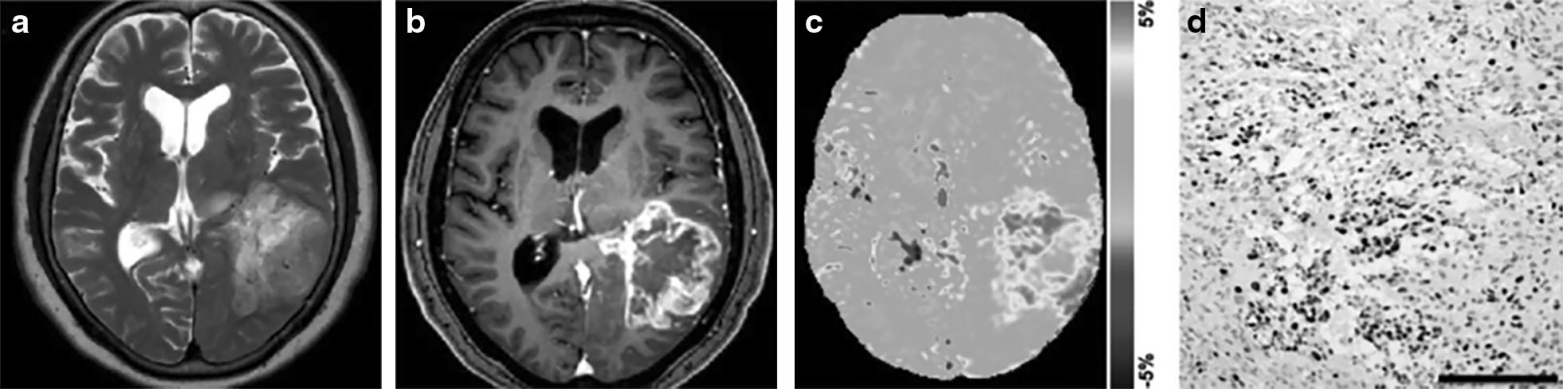
APT imaging of brain tumor (glioblastoma multiforme) in a human patient. Anatomical T2 weighted (**a**) and post contrast T1 weighted (**b**) images are showing diffuse tumor in parietal lobe. APT weighted image (**c**) shows high contrast in tumor than normal brain parenchyma. Immunohistochemical staining of Ki-67 (**d**) shows very high proliferative activity in tumor with high cellular density. Reproduced with permission from the Oxford University Press and Togao et al. [83]

**Fig. 4:**
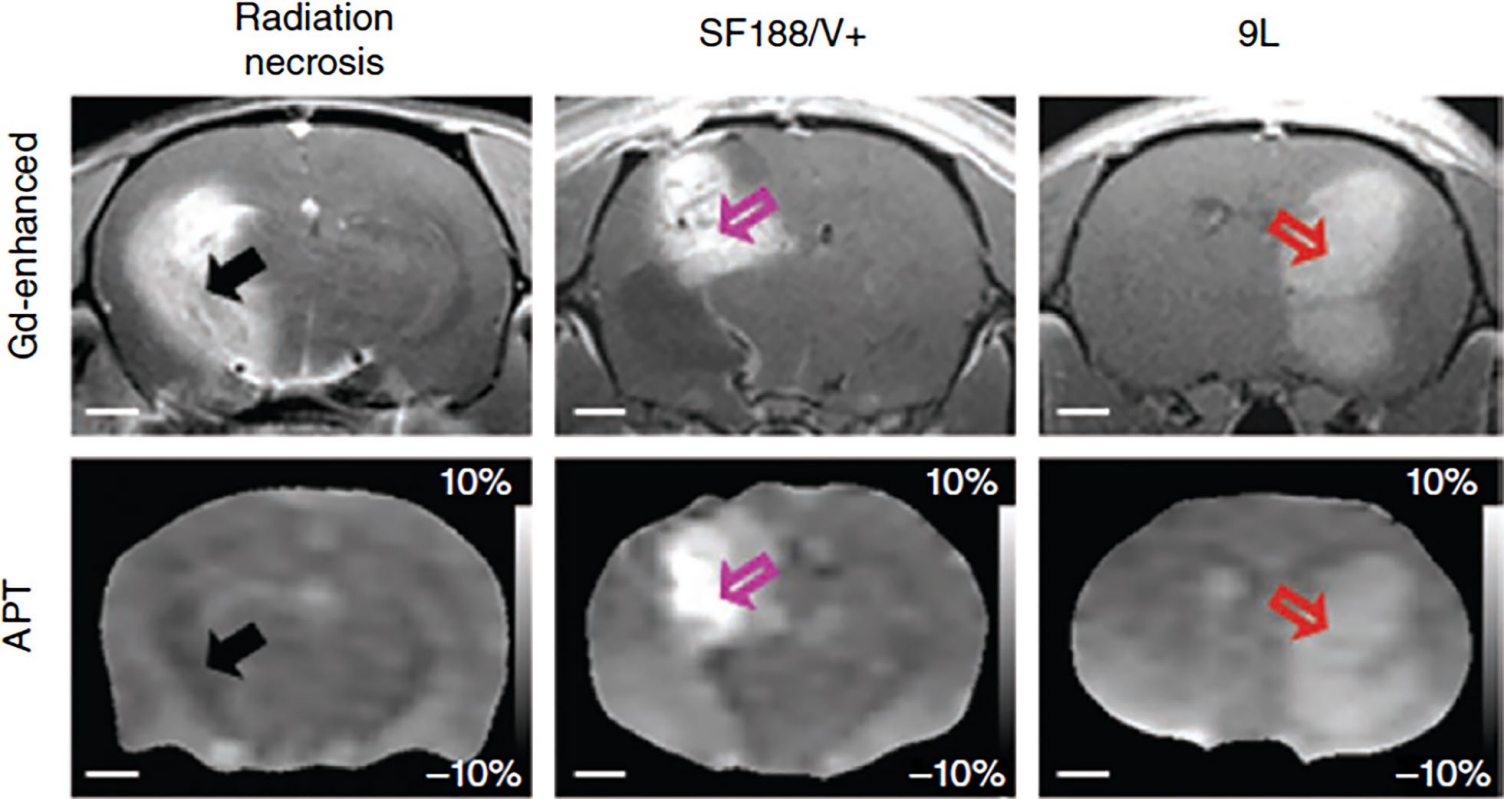
Differentiation of radiation necrosis and glioma using APT MRI. MRI of radiation treated animals is performed after 178 days of 40 GY radiation treatment. Radiation necrosis (*black arrow*) area as revealed by Gd enhancement shows hypointense to isointense on APT weighted image compared to contralateral brain tissue. While both SF188/V + (*pink arrow*) and 9L (*red arrow*) tumors show hyperintensity both on the Gd enhanced and APT-weighted images, which correspond to high cellularity. Reproduced with permission from the Nature Publishing Group and Zhou et al. [89]

Amide proton transfer (APT) imaging is one of the widely used CEST based methods which is being used to image mobile protein and peptides in cancers in vivo [82–86]. Briefly, APT imaging method depends on exchange between protons of free water and those of amide groups (–NH) of endogenous mobile proteins and peptides [87]. Higher numbers of such amide protons are reported in cancer compared to normal healthy tissue. APT imaging of the human brain tumor showed higher APT contrast in tumor region than contralateral normal brain parenchyma [83, 88–90]. It has been shown that APT can better discriminate tumor from edema and normal brain areas than conventional T2, T1 and FLAIR imaging [88]. APT imaging has been used to classify the tumors, which showed higher APT contrast in high grade tumor compared to low grade tumor [83] (Fig. 3). In a recent study, APT imaging has been used to distinguish tumor recurrence from radiation necrosis [89]. While the conventional methods cannot reliably differentiate between tumor recurrence and radiation necrosis, APT contrast was shown to be hyper-intense in tumor tissues and hypo-intense in necrotic areas (Fig. 4) [89]. APT was also used to evaluate the radiation treatment monitoring in cancer, which showed decreased APT contrast post radiation treatment [89].

**Fig. 5:**
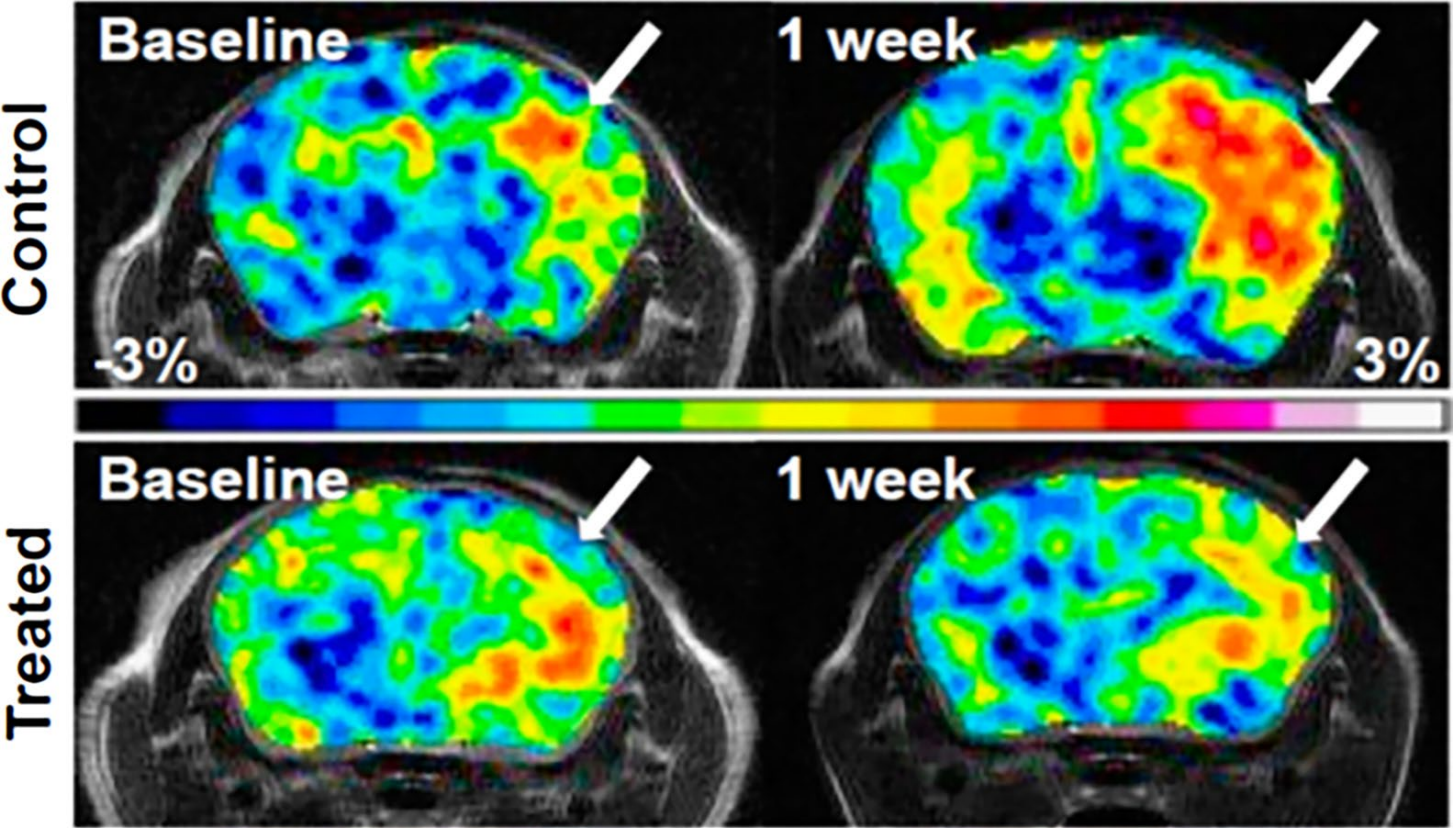
Monitoring TMZ treatment response in mouse model of human glioblastoma multiforme using APT contrast. APT weighted images are showing increased APT contrast in control animal than treated animal. Lower contrast in treated group corresponds to decreased tumor cells proliferation. Reproduced with permission from the National Academy of Sciences and Sagiyama et al. [90]

Amide proton transfer weighted imaging has been also used to assess the early treatment response during short-term chemotherapy with temozolomide (TMZ) in mouse model of glioblastoma multiforme (GBM), which showed decreased APT contrast following treatment, while in control non-treated group increased APT contrast is observed [90] (Fig. 5). The treated group showed low level of Ki67, an index of tumor cells proliferation, than nontreated control tumor (Fig. 5). In another experiment, TMZ-resistant GBM line showed increase in both APT signal and levels of Ki67 despite the same course of TMZ treatment [90]. Based on these observations it has been suggested that the APT signal may be useful in monitoring the treatment response and to evaluate the tumor progression.

**Fig. 6:**
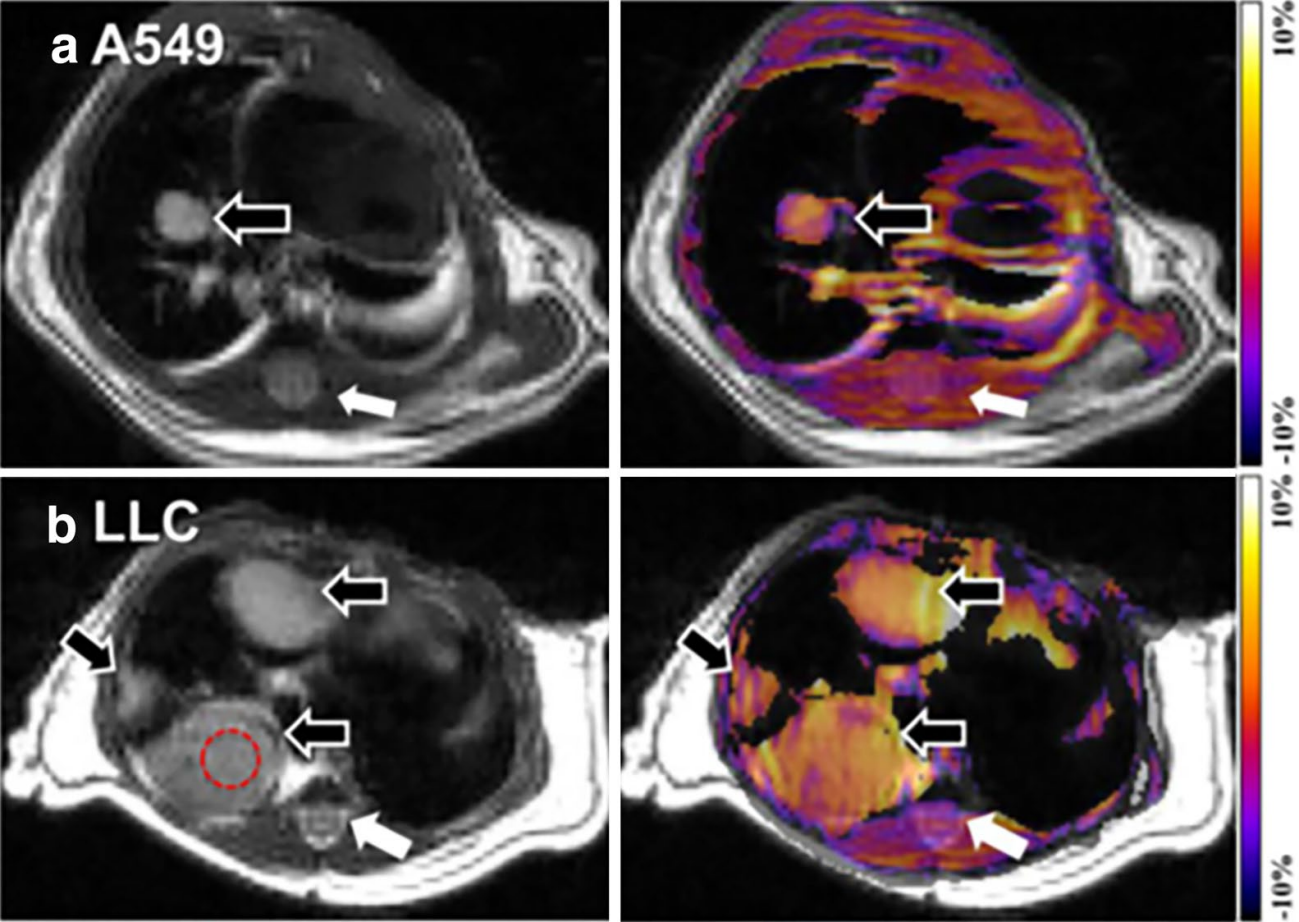
APT imaging of lung tumors. Anatomical proton weighted image and APT-weighted images of A549 (**a**) and LLC (**b**) tumors in mouse model. Both the tumors showed higher APT contrast than surrounding tissues including spinal cord (*white arrows*) and skeletal muscles. Higher CEST contrast is detectable on LLC tumor than A549 tumor (*black arrow*). Reproduced with permission from Public Library of Science and Togao et al. [84]

In a very recent study, APT was used to image orthotropic lung tumor in mouse model and has showed higher APT contrast in tumor [84] (Fig. 6). It is presumed that APT can be used as a potential biomarker to characterize and grade the lung cancer non-invasively in vivo. Applications of APT imaging are emerging in studies of other cancer types including breast cancer, prostate cancer, bladder cancer etc.

**Fig. 7:**
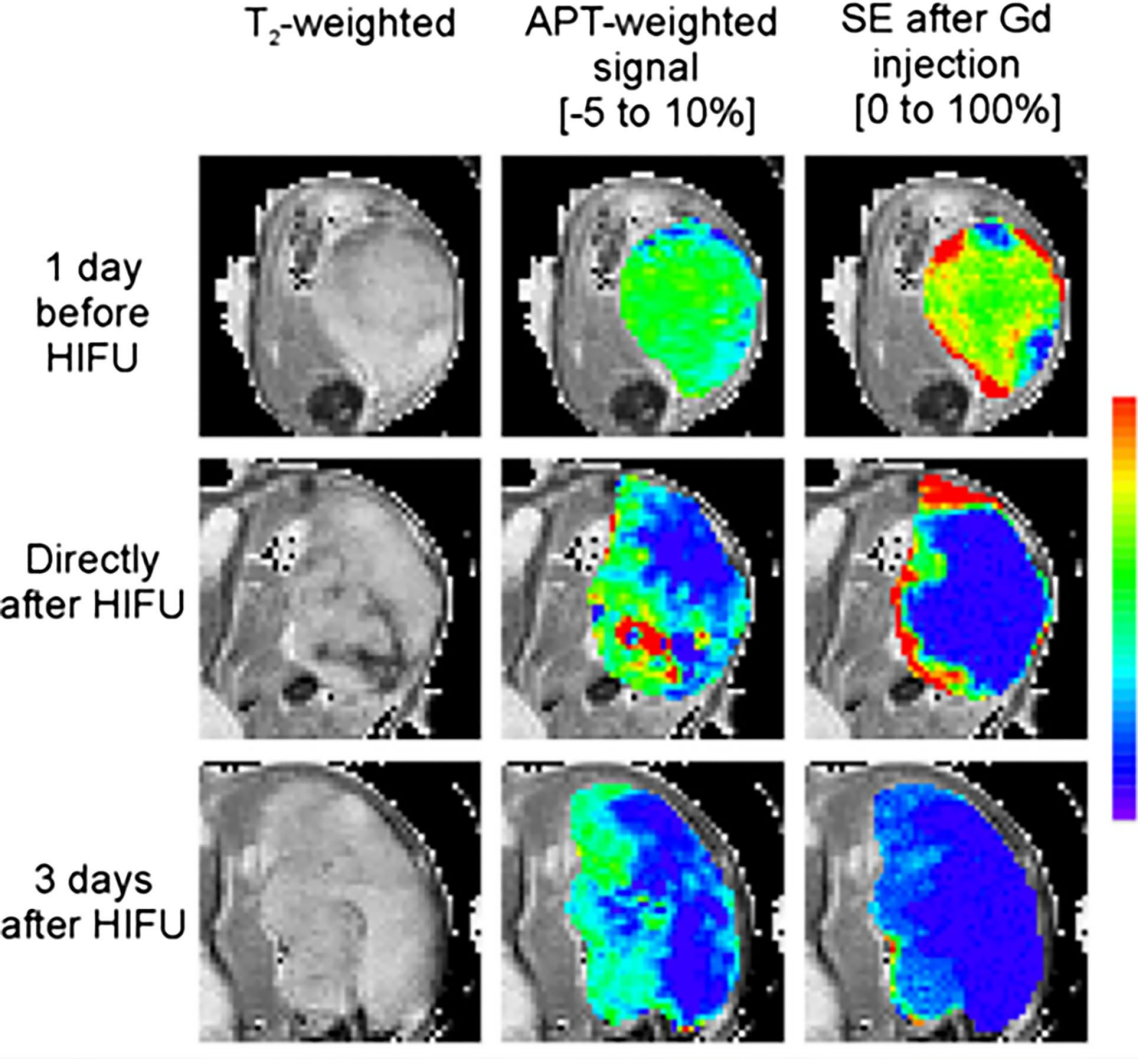
Monitoring HIFU treatment in cancer. Proton anatomical image, APT weighted image and GD contrast enhanced image show the changes in tumor following HIFU treatment. Decreased APT contrast following HIFU was observed which is comparable to the Gd based contrast enhancement study. Reproduced with permission from John Wiley and Sons and Hectors et al. [91]

Treatment response to high intensity focused ultrasound (HIFU) in animal model of tumor has been monitored through APT weighted imaging [91]. A decrease in APT contrast in tumor following HIFU treatment was observed, which was complementary to the gadolinium (Gd) based study [91] (Fig. 7). This suggests that APT can effectively replace the Gd based monitoring of HIFU treatment response in cancer. Moreover, APT uses endogenous mobile proteins and peptides for signal measurement while in Gd based study exogenous administration of contrast is required. Despite plethora of applications, the physiological basis of APT and APT changes are still not well understood.

### CEST imaging of small metabolites in tumor

Role of different biochemical signatures in cancer malignancy is known since decades. Efforts have been made to quantify changes in these metabolites non-invasively using ^1^H MRS, which showed altered concentration of different metabolites in tumor tissue [26]. These metabolites include glutamate, creatine, myo-inositol, glycine, etc. However, ^1^H MRS suffers from poor resolution and does not provide information regarding heterogeneous distribution of metabolites concentration in cancer tissue. Recently, high resolution imaging of these metabolites has been performed using CEST imaging [92–94]. However, changes in pH and other biological factors may affect the quantification of CEST contrast from these metabolites in cancer. Most of these metabolites present intracellular in cancer, and it is well known that the intracellular pH in cancer mostly does not change, therefore, by using CEST method concentration changes of these metabolites in response to tumor aggressiveness can be quantified and may be used as a bio-marker to evaluate therapeutic responses.

**Fig. 8:**
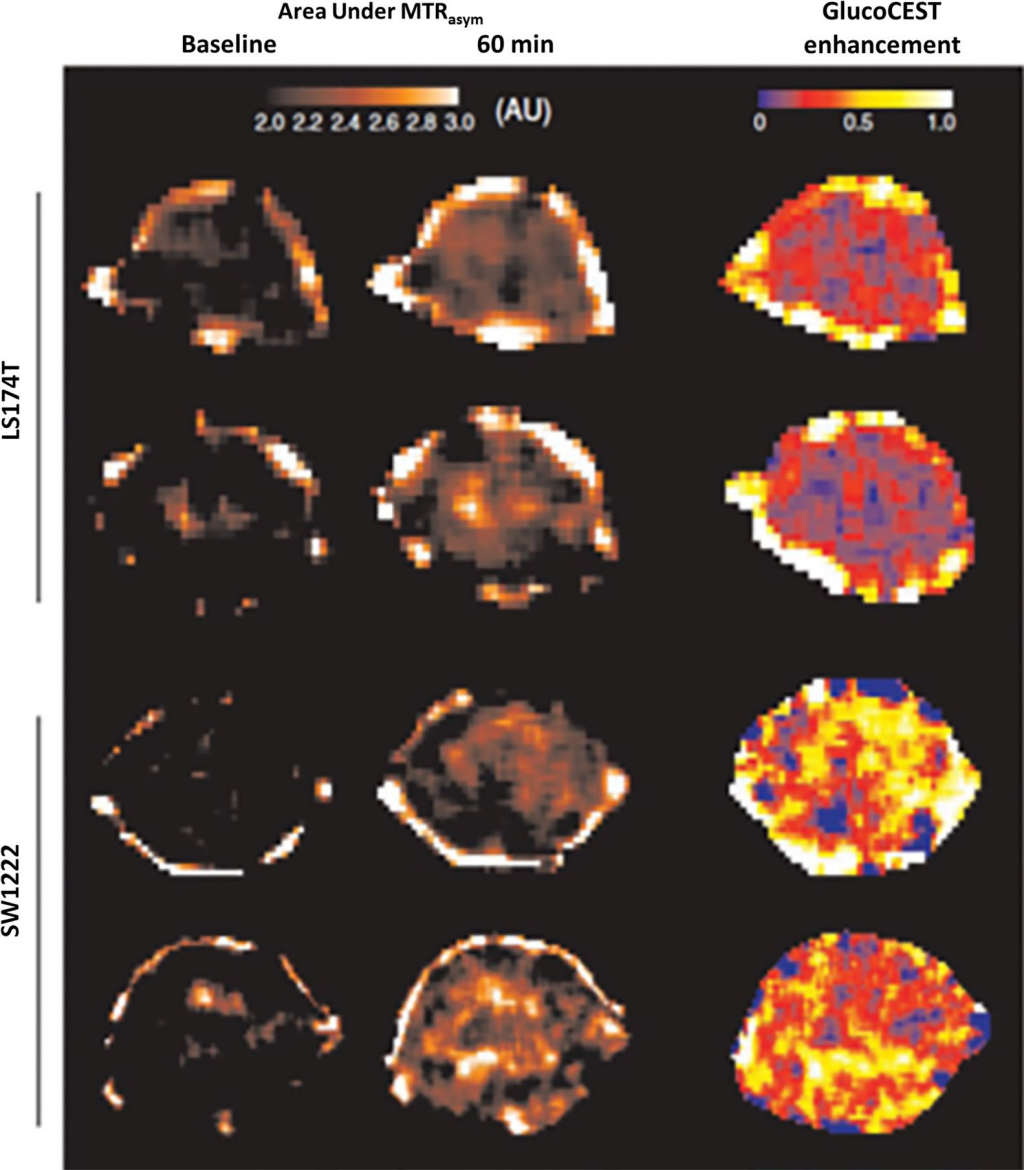
GluCEST imaging in mouse model of colorectal cancers (SW1222 than LS174T). SW1222 cancers show higher GluCEST contrast than LS174T cancers. Reproduced with permission from the Nature Publishing Group and Walker-Samuel et al. [95]

Recently, glucose was used as exogenous CEST contrast agent to monitor the glucose CEST enhancement (GCE) in mouse model of colorectal cancers [95]. Higher GCE was observed in SW1222 than LS174T cancer (Fig. 8) [95]. Based on the findings, it was further suggested that the GCE can be used to distinguish tumor types with differing phenotypic characteristics. Since glucose is not toxic and is readily available it can be rapidly used in clinic to evaluate the different types of cancers.

**Fig. 9:**
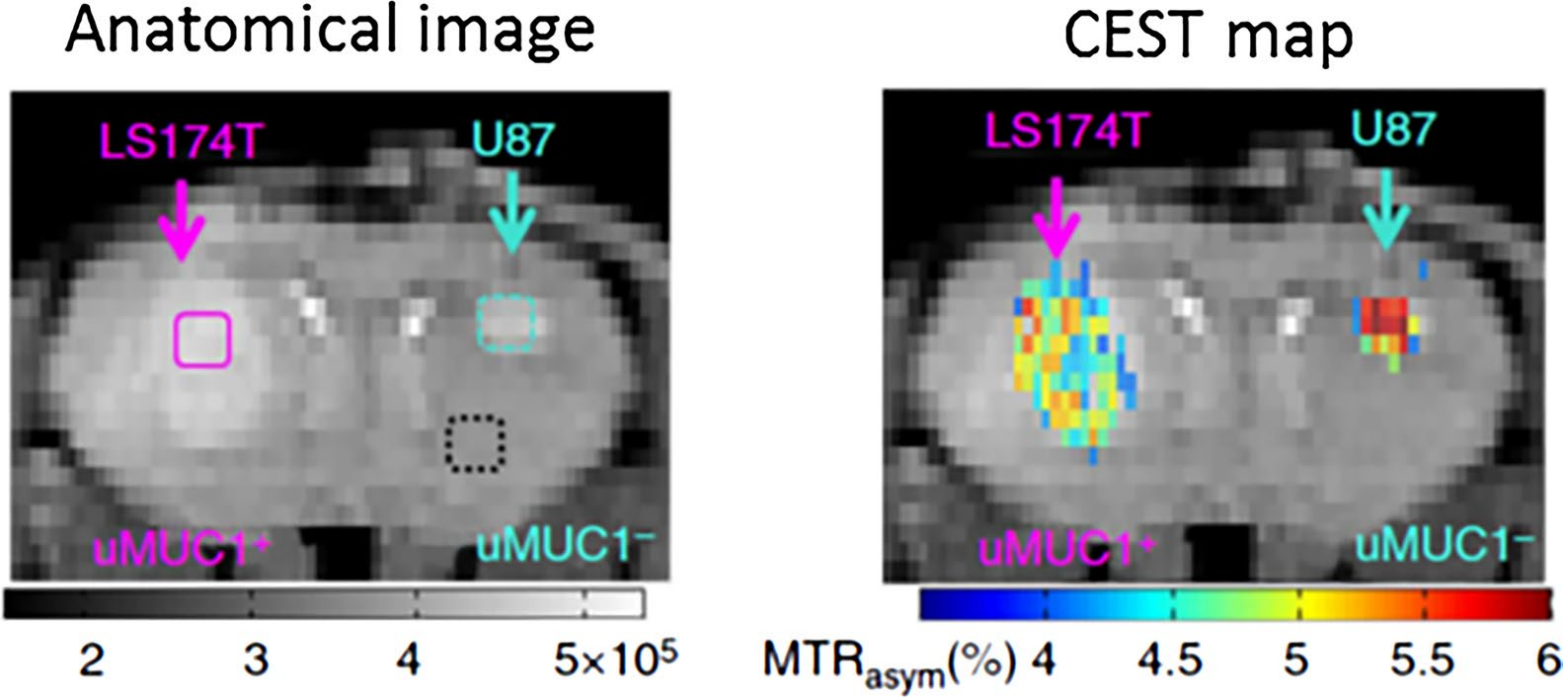
Mucin dependent CEST contrast imaging. Underglycosylatedmucin 1 (uMUC1+) overexpressed in LS174T which shows significantly low CEST contrast compared to the U87 tumor which is devoid of mucin expression. Reproduced with permission from the Nature Publishing Group and Song et al. [97]

Alterations in mucin expression and glycosylation are associated with the cancer progression and invasion [96]. Very recently CEST technique has been used to differentiate the glycosylated mucin tumor from underglycosylated mucin tumor, and showed that the deglycosylation of mucin resulted in more than 75 % reduction in CEST contrast [97] (Fig. 9).

### CEST imaging of protease enzyme expression

**Fig. 10:**
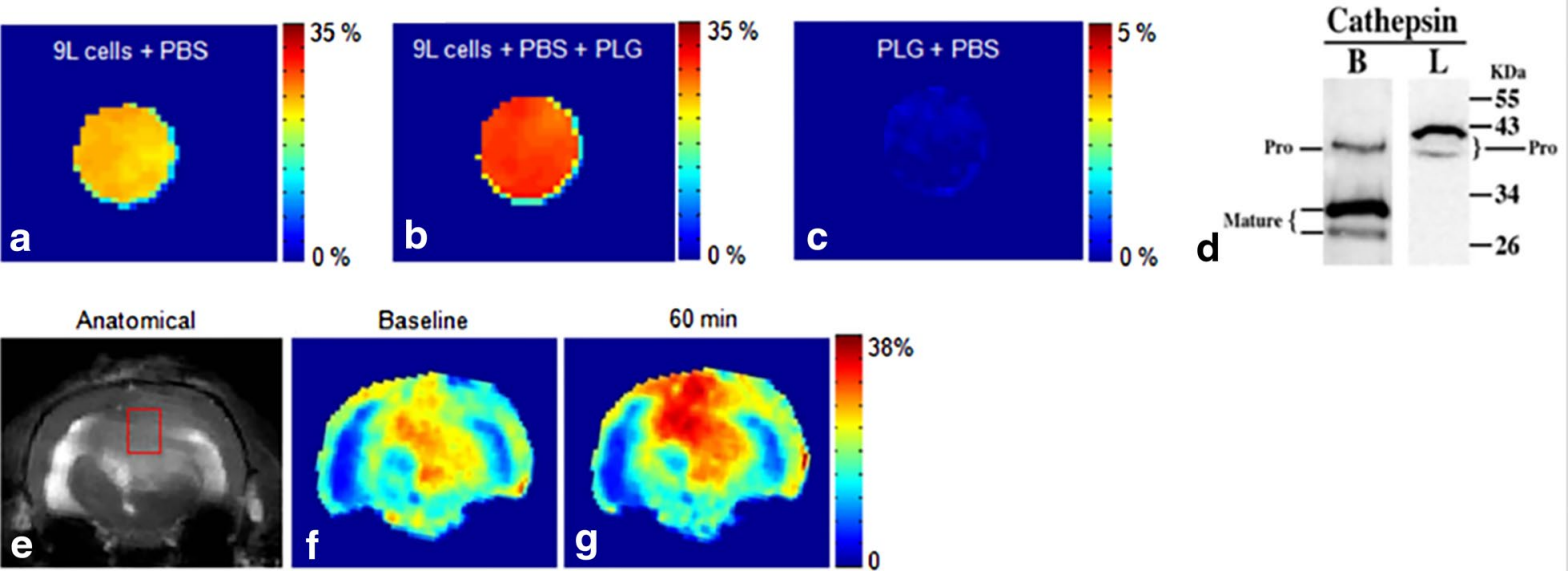
CEST imaging of protease enzyme expression in cancer. **a** GluCEST map of 9L cancer cells cultured without PLG. **b** GluCEST map of 9L cell line cultured in the presence of PLG showed ~17 % increased GluCEST contrast compared to (**a**), which is due to the cleavage of PLG by CtB present in the tumor cells. **c** PLG solution in PBS does not show any appreciable GluCEST contrast. **d** Western blot analysis shows expression of cathepsin B in both mature and pro form while cathepsin L only in the pro form. The cleavage of PLG in this tumor cell line is predominantly due to CtB. **e**, **f** Anatomical image and GluCEST map from a rat brain with a 9L tumor. **g** At 60 min post intra-venous injection of PLG increased GluCEST contrast was observed in the tumor region due to cleavage of PLG possibly by proteases. Reproduced with permission from the Nature Publishing Group and Haris et al. [100]

Developing proteolytic enzyme inhibitors is an active area of research and the ability to non-invasively detect proteolytic enzyme activity would be valuable in selecting tumors for specific inhibitors and for detecting response to such agents. MRI Studies have been performed to detect the transglutaminase [98] and Hyaluronidase [99] activity in preclinical setup using contrast generated by a peptide linked to GdDTPA. Recently, Haris et al., have used GluCEST method to image the cathepsin protease activity in 9L tumor in rat model using poly-L-glutamate as a CEST imaging probe [100] (Fig. 10). Cathepsin protease expressions in tumor cleave the PLG into smaller fragments or its monomers and expose several glutamate amine protons, which can be monitored noninvasively using GluCEST technique. Since the elevated protease activity is highly associated with tumor malignancy [101], by monitoring the kinetics of poly-L-glutamate (PLG) cleavage tumor aggressiveness can be mapped. Further, PLG has been used as a macromolecule for the targeted cancer drugs delivery [102], this method can be potentially used to monitor targeted drug delivery as well as their efficacy on tumor cells. This technique may also provide a novel diagnostic tool for early detection of tumors and in effective anti-cancerous drug designing.

### CEST imaging of cancer tissue redox potential

**Fig. 11:**
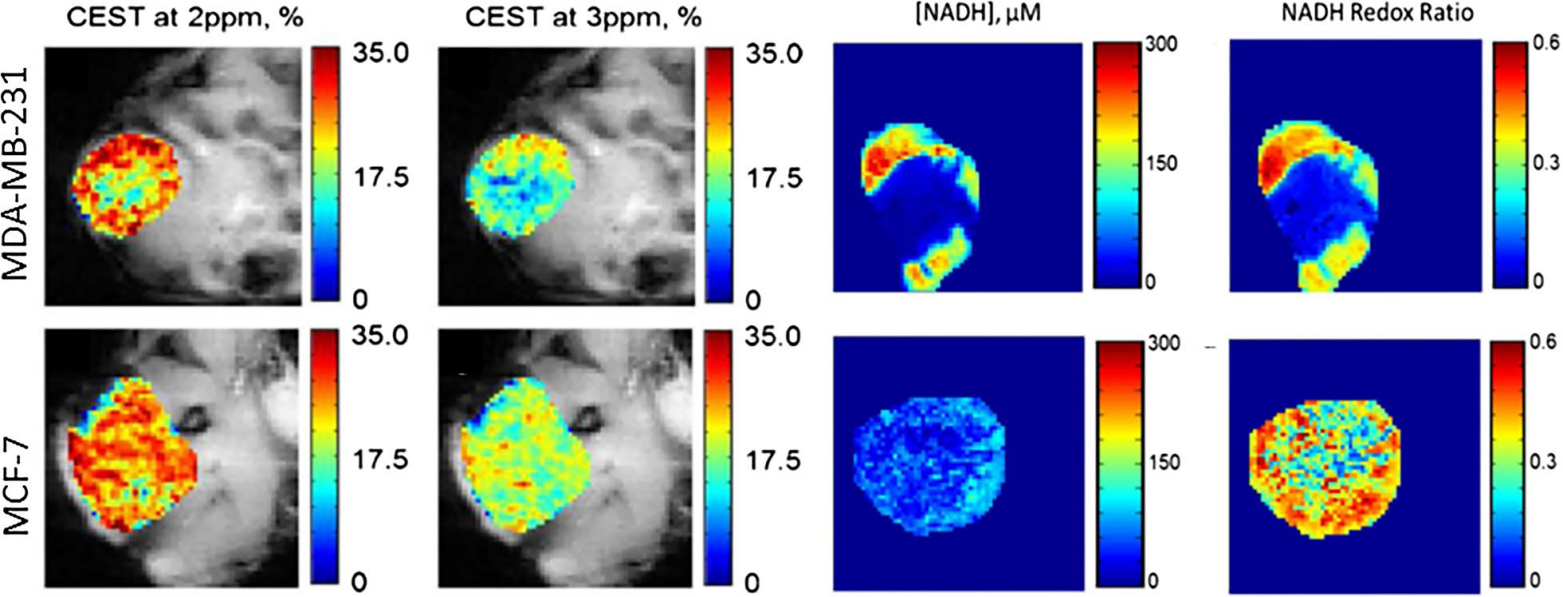
CEST imaging of breast cancer. CEST maps (at 2 and 3 ppm) of flank MDA-MB-231 and MCF-7 mice breast cancers models. NADH and NADH redox ratio show higher rim to core ratio of NADH concentration in MDA-MB-231 than MCF-7 tumor. Reproduced with permission from Springer and Cai et al. [104]

Tumor redox potential is an important marker to predict the biological as well as metabolic processes in tumor cells [103]. The tumor redox state has been recently imaged through CEST MRI in two different breast cancer mouse xenograft models, and correlated with the redox measurement by optical imaging method [104] (Fig. 11). Cai et al. have observed that the CEST contrast from tumor tissue linearly correlated with the Nicotinamide adenine dinucleotide (NADH) concentration as well as NADH redox state. Cai et al. [105] have further extended the CEST methods to characterize the prostate cancer in vivo in preclinical mouse model. Significantly higher CEST contrast was observed in PC-3 than DU-145 prostate cancer [105].

The above studies suggest that once validated, the CEST methods can be used in clinical setup to characterize tumor aggressiveness and to monitor the therapeutic responses in vivo.

### MRI reporter genes in cancer

Gene expression in cancer cell can be monitored using reporter genes. Expression of reporter genes such as beta-galactosidase and luciferase has been examined ex vivo using immunohistochemical and histological techniques. Though, ex vivo methods provide high specificity and sensitivity, they cannot provide dynamic information, and require large number of animals to sacrifice for a longitudinal study. On the other hand, optical imaging can provide some structural and functional information about the gene expression in vivo but the sensitivity deteriorate very fast in the deeper part of tissue. These problems have been overcome by developing MR based reporter genes, which enable in vivo imaging of cell proliferation and migration.

**Fig. 12:**
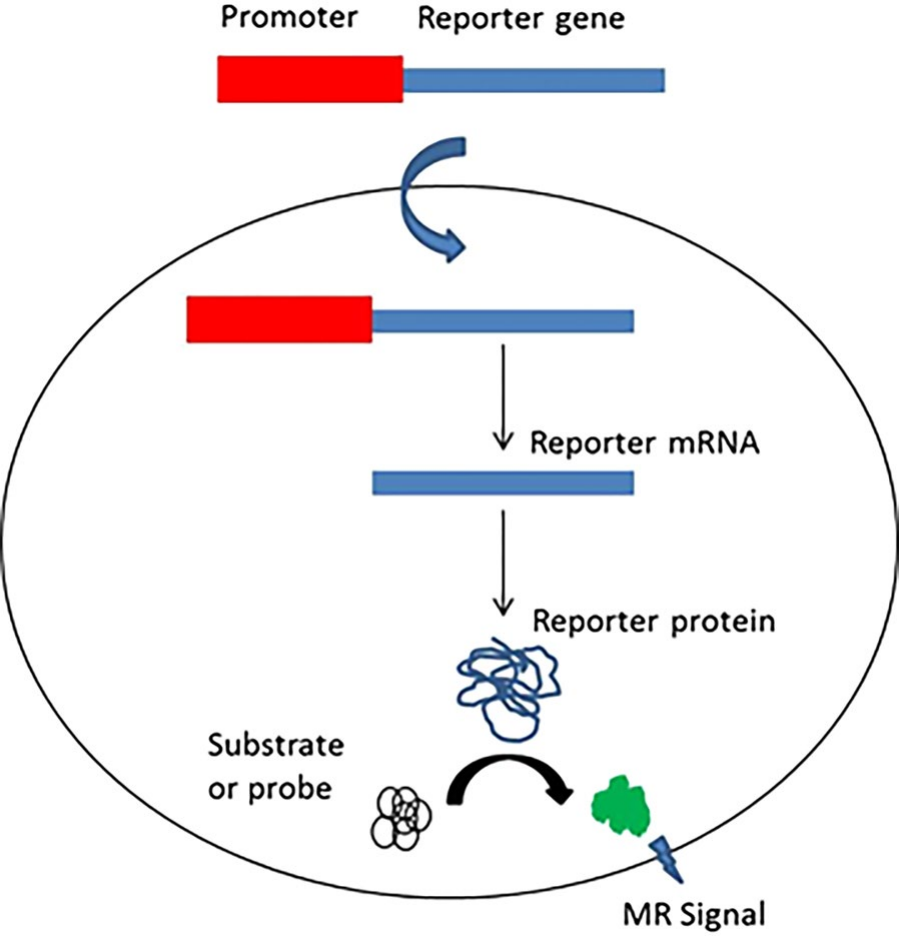
Reporter gene imaging. Reporter gene inserted in the downstream of a gene promoter. The promotor activation transcribes reporter gene to reporter mRNA, which produces reporter protein after translation. The reporter protein converts the substrate or probe into active form which can be imaged using MRI

Magnetic resonance reporter genes have potential to monitor the transgene expression in vivo noninvasively. These genes can be applied to interrogate the efficacy of gene therapy, to assess cellular differentiation, cell trafficking, and specific metabolic activity, and also assess changes in the microenvironment [106]. Various MR reporter genes probes are generated to monitor the activity of different genes in biologic systems in vivo. Reporter genes basically generated by fusing promoter from a gene of interest to the gene of either a easily detectable protein or an enzyme capable of generating detectable contrast agent upon reaction with its substrate (Fig. 12). Gene expression imaging has had a revolutionary impact on laboratory study of cancer biology and is likely to play an important role in clinical trials in the future.

**Fig. 13:**
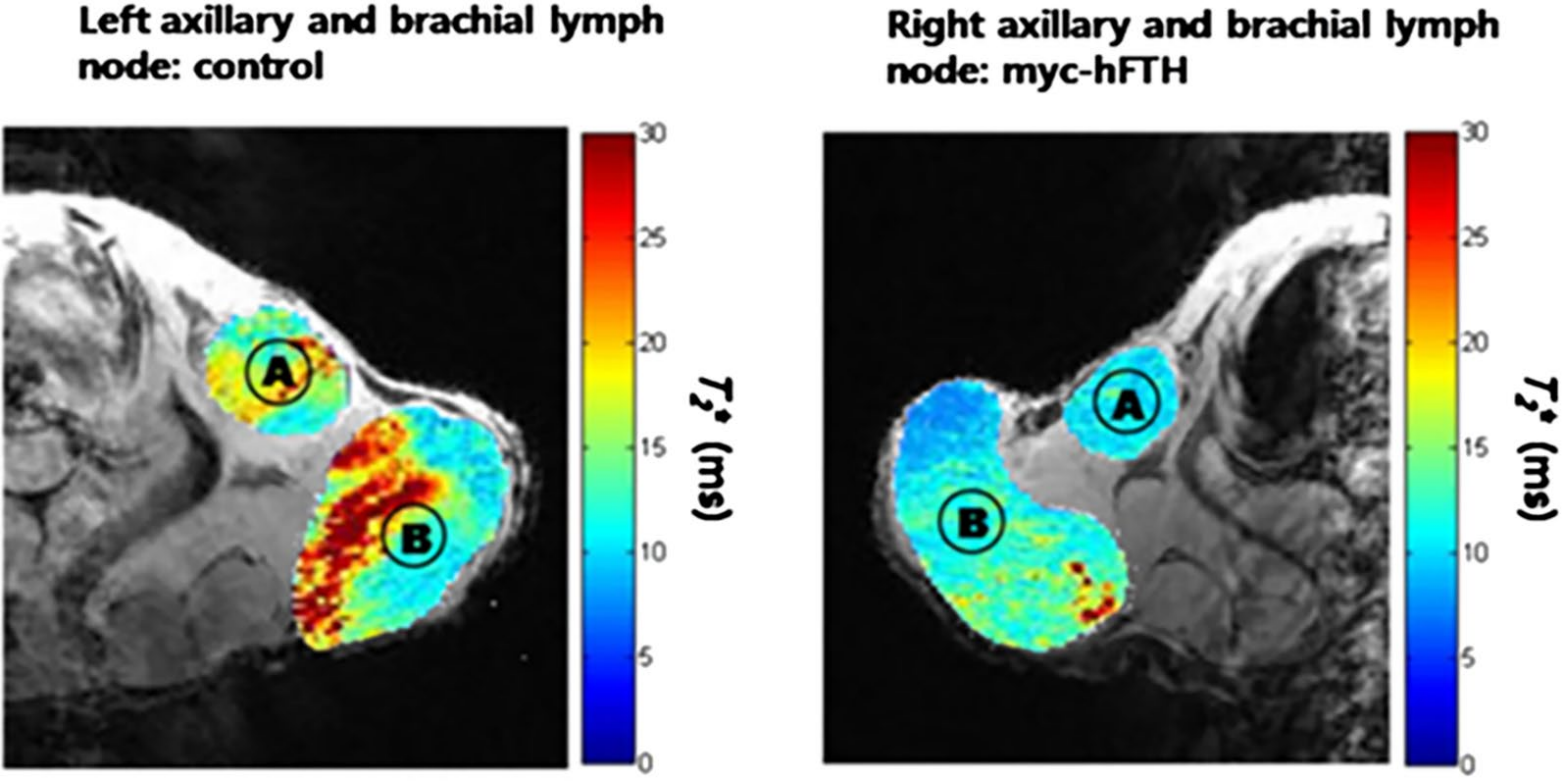
In vivo imaging of metastatic cells expressing myc-tagged human ferritin heavy chain (myc-hFTH) in lymph nodes (LNs). T_2_* map of metastasis from control and myc-hFTH cells in the left and right axillary (*A*) and brachial (*B*) LNs in nude mice. Reproduced with permission from John Wiley and Sons and Choi et al. [107]

Ferritin MR reporter gene has been used to track the metastatic melanoma cells in lymph node [107] (Fig. 13) and to image the C6 glioma tumor cells [108] in vivo in preclinical mouse model. The cancer cells expressing the human ferritin protein can be detected as low signal intensity both on T_2_ and T_1_ relaxation weighted images. The change in the T_2_ and T_1_ relaxation properties can be used to track cancer cells in vivo as well as to evaluate the therapeutic effect of different drugs on cancer cells. In one of the study, ferritin high chain (FHC) overexpressing fibroblasts administered intraperitonealy in a mouse model of human ovarian cancer [109]. The recruitment of fibroblasts was monitored using R_2_ (1/T_2_) mapping of tumor which demonstrated high R_2_ value at the tumor rim compared to the tumor mouse which received control fibroblasts [109].

**Fig. 14:**
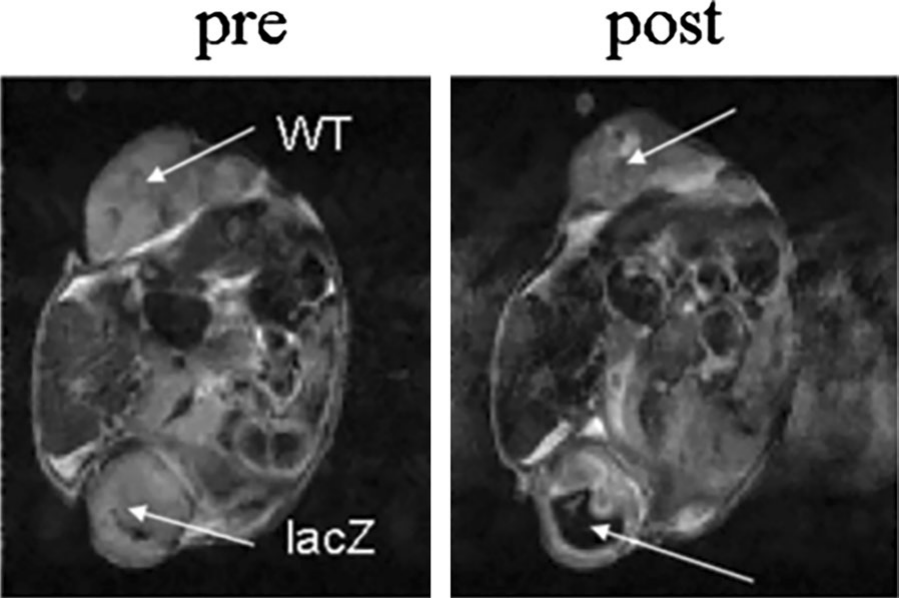
MRI detection of beta b-gal activity in MCF breast tumor transfected with lac Z. After intratumoral injection of S-Gal and ferric ammonium citrate, the tumor expressing lac Z shows strong hypointense contrast [111]. Reproduced with permission from John Wiley and Sons and Cui et al. [111]

Arena et al. [110] have used the Lac Z as an MRI reporter gene to image proliferation of mouse melanoma cells. The lac Z expressing tumor cells can be easily distinguished by exogenous administration of a gadolinium based contrast agent. This gadolinium based contrast was designed as such that it maintain gadolinium ion in a water inaccessible position until it cleaved by the β-galactosidase enzyme express by the Lac Z. The cleavage results in transition of Gd ion in a water accessible position which generate strong positive contrast on T_1_ weighted MRI. Cancer cells expressing the Lac Z provides higher contrast than control after exogenous administration of contrast agent. In another study, the interaction between β-gal and a staining salt i.e. 3,4-Cyclohexenoesculetin b-D-galactopyranoside in presence of ferric ions generated a strong hypointensity on T_2_* weighted image in tumor cells expressing LacZ [111] (Fig. 14).

**Fig. 15:**
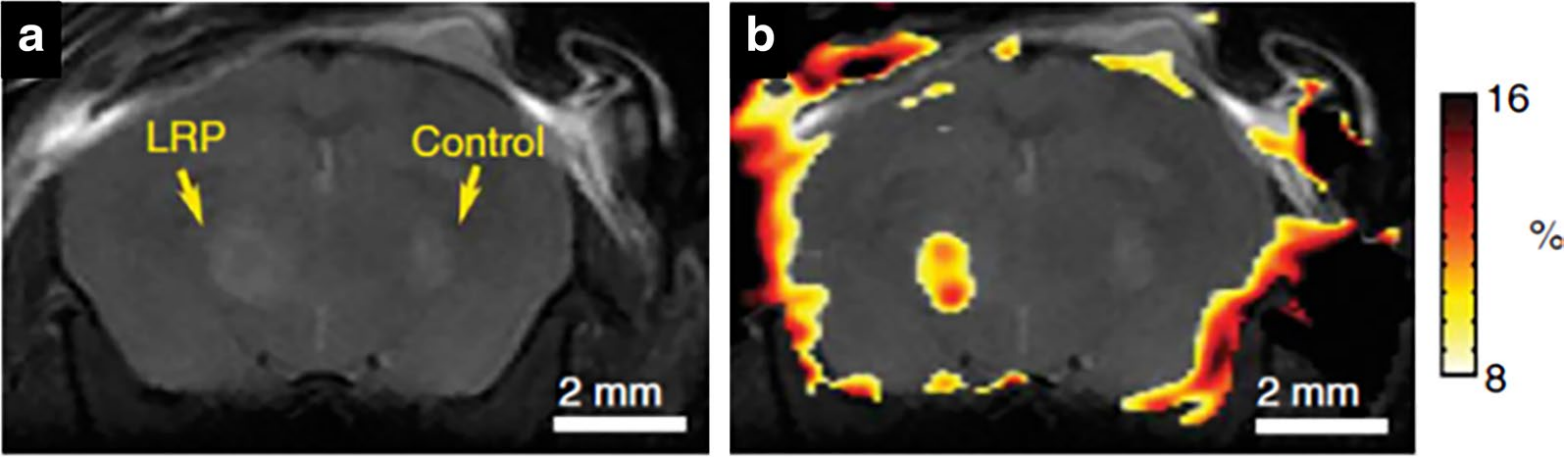
Lysine rich protein (LRP) based MR reporter genes transfected with the 9L gliosarcoma cells before implantation in the rat brain. On anatomical imaging both the LRP and control xenografts show the similar signal intensity (**a**). The CEST map highlighted the LRP xenograft due to the expression of lysine rich protein, which can be easily detected through APT CEST (**b**). Reproduced with permission from the Nature Publishing Group and Gilad et al. [112]

With exception of ferritin all other MR reporter genes are rely on administration of substrate, which limits it’s access to all tissues. Poly-l-lysine ability to use as a CEST contrast provided the concept for the synthesis of an artificial gene rich in lysine residue [112]. The 9L rat glioma cells’ overexpressing this transgene is easily distinguished from the control 9L cells tumor (Fig. 15) [112].

### Molecular MR imaging in cancer immunotherapy

Despite significant advances in chemotherapy and radiotherapy, cancer treatment remains an immense challenge. Cell-based cancer immunotherapy is gaining widespread attention as it provides a novel approach to treat cancer by triggering the patients’ own immune system to induce a potent anti-tumor response. Various cell types, such as lymphocytes (CD4+ and CD8+ T cells), dendritic cells (DCs) and natural killer cells (NK cells) have shown their therapeutic efficacy to treat cancer patients. This provides a highly selective way to kill cancerous cells with significantly less side effects on normal cells.

Currently, the only way to monitor the bio-distribution and pharmacokinetics of these therapeutic cells is rely on immunohistochemistry of the excised tissues. Monitoring delivery and therapeutic effect of these cells in vivo non-invasively can make the difference in failure and success to cancer immunotherapy. Non-invasive monitoring of the disposition, migration and destination of therapeutic cells will facilitate the development of cell based therapy. In general, response to immunotheryapy is mostly evaluated by monitoring alteration in tumor size, tumor markers and improved survival rates, which require several weeks to months or even years for the treatment assessment. The localization of immune cells at the tumor site is an early marker for the treatment response, which is primarily examined through invasive histologic tissue analyses.

With the development in the molecular imaging technology it is possible to track the fate of these therapeutic cells in vivo non-invasively. Noninvasive molecular imaging has potential to provide instant assessment of cell based therapy in both clinical and preclinical settings. These immunotherapeutic cells can be modified as such so that they can be easily detectable by MRI by introducing imaging probes into cells or attaching them on cell surface before injection. The obtained imaging signals can potentially be used as biomarkers for tumor response and for differentiating patients who are responders or nonresponders to immunotherapy.

In a recent study, DCs have been directly labeled using ^19^F, perfluoropolyether (PFPE) which can be visualized by MRI [113]. It is observed that PFPE have insignificant effect on DC function and moreover, by using ^19^F more selective images can be obtained as the background signal for ^19^F within tissues is negligible. With the advent of the iron oxide nano particles and their approval from FDA in clinical use open a new approach to detect the infiltrating immune cells to tumor cells in vivo. Cells labelled with nanoparticles produce strong hypointense contrast on MRI and showed promise of direct clinical translation. Sheu et al. [114] showed that tumor signal changes in T_2_* relaxation time maps generated from gradient echo sequences after intra-arterial infusion of SPIO-labeled NK-92cells.

To overcome the toxicity effect to normal cells, in a very recent approach cancer natural killer (NK) cells are genetically altered to recognize tumor associated surface antigens, which showed highly efficient toxicity against tumor cells with minimal or no effect on normal healthy cells. Different tumor associated antigens have been recognized as target for the NK cells- for example ErbB2/HER2 receptor tyrosine kinase in breast and ovarian cancer cells, pan-carcinoma antigen epithelial cell adhesion molecule (EpCAM) in prostate cancer cell, and CD20 differentiation antigen in B cell malignancies.

**Fig. 16:**
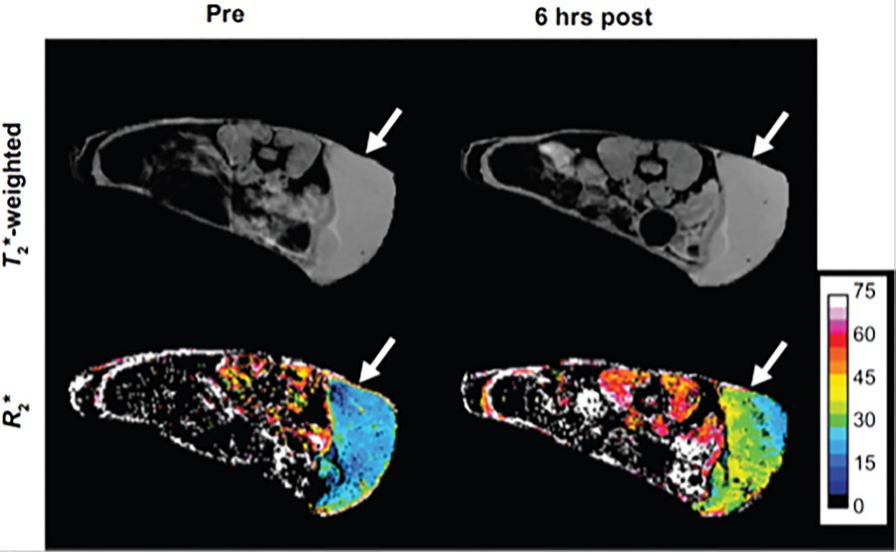
Tracking the NK cells in mouse model of Daudi Burkitt’s lymphoma. Axial T_2_*-weighted gradient echo images show the flank tumor (arrow). The corresponding R_2_* maps show increase in R_2_ signal 6 h post ferumoxytol-labeled NK cells injection. Reproduced with permission from Thomas Hill Publisher and Sta Maria et al. [116]

EpCAM-targeted and ferumoxide labeled NK cells demonstrated substantial decreased T_2_* signals in EpCAM-positive prostate cancers than ferumoxide labeled nontargeted parental NK-92 cells [115]. The relaxation maps R_2_* (R_2_* = 1/T_2_*) can be generated and used to quantify labeled cells in tumor and other tissues. Figure 16 is showing a T2*-weighted image and the corresponding R2* map of a Daudi Burkitt’s lymphoma in a mouse model before and 6 h post injection of ferumoxtyol-labeled NK cells, which is depicting higher R_2_* signal in the tumor region [116]. MRI can provide a noninvasive and real-time immune cell tracking which may provide a surrogate marker for tumor response as early as 24 h after start of therapy.

## Conclusions

Molecular MR imaging is well suited to measure molecular and cellular processes including metabolism, apoptosis, cell proliferation and biosynthetic pathways of different metabolites in vivo in cancer. Molecular imaging can play an important role in every aspect of oncology practice, including early disease detection, diagnosis, staging, personalized treatment, and treatment monitoring. Prostate, ovarian and lung cancer are just a few of the many types of cancer in which molecular imaging truly changed the direction and outcome of the patient care. The ability of molecular imaging to detect abnormalities very early in the progression of disease has the potential to change medicine from reactive to proactive, detecting and curing disease in its most treatable phase and saving countless lives. In medical setup, molecular MRI will pave the way toward a significant improvement in early detection of disease, therapy planning and monitoring the therapeutic outcomes.

## Abbreviations

MR: magnetic resonance
SPECT: single photon emission computed tomography
PET: positron emission tomography
MRI: magnetic resonance imaging
PPP: pentose phosphate pathway
MRS: magnetic resonance spectroscopy
^31^P: phosphorus
^13^C: carbon
^19^F: fluorine
^1^H MRS: proton MR spectroscopy
Cho: choline
NAA: N-acetylaspartate
PC: phosphocholine
GPC: glycerol-PC
MRSI: magnetic resonance spectroscopic imaging
NTP: nucleotide triphosphate
PME: phosphomonoesters
PDE: phosphodiesters
Pi: inorganic phosphate
CEST: chemical exchange saturation transfer
APT: amide proton transfer
TMZ: temozolomide
GBM: glioblastoma multiforme
HIFU: high intensity focused ultrasound
GCE: glucose CEST enhancement
PLG: poly-L-glutamate
NADH: nicotinamide adenine dinucleotide
FHC: ferritin high chain
PFPE: perfluoropolyether
SPIO: superparamagnetic iron oxide
NK: natural killer
EpCAM: epithelial cell adhesion molecule
